# Formation Mechanism of Benzo(a)pyrene: One of the Most Carcinogenic Polycyclic Aromatic Hydrocarbons (PAH)

**DOI:** 10.3390/molecules24061040

**Published:** 2019-03-15

**Authors:** Edina Reizer, Imre G. Csizmadia, Árpád B. Palotás, Béla Viskolcz, Béla Fiser

**Affiliations:** 1 Institute of Chemistry, University of Miskolc, Miskolc-Egyetemváros, H-3515 Miskolc, Hungary; reizeredina@gmail.com (E.R.); icsizmad@hotmail.com (I.G.C.); bela.viskolcz@uni-miskolc.hu (B.V.); 2 Department of Chemisrty, University of Toronto, Toronto, M5S 1A1 Ontario, Canada; 3 Institute of Energy and Quality Affairs, University of Miskolc, Miskolc-Egyetemváros, H-3515 Miskolc, Hungary; arpad.palotas@uni-miskolc.hu; 4 Ferenc Rákóczi II. Transcarpathian Hungarian Institute, UA-90200 Beregszász, Transcarpathia, Ukraine

**Keywords:** PAH, growth mechanism, DFT, chrysene, benz(a)anthracene, benzo(a)pyrene

## Abstract

The formation of polycyclic aromatic hydrocarbons (PAHs) is a strong global concern due to their harmful effects. To help the reduction of their emissions, a crucial understanding of their formation and a deep exploration of their growth mechanism is required. In the present work, the formation of benzo(a)pyrene was investigated computationally employing chrysene and benz(a)anthracene as starting materials. It was assumed a type of methyl addition/cyclization (MAC) was the valid growth mechanism in this case. Consequently, the reactions implied addition reactions, ring closures, hydrogen abstractions and intramolecular hydrogen shifts. These steps of the mechanism were computed to explore benzo(a)pyene formation. The corresponding energies of the chemical species were determined via hybrid density funcional theory (DFT), B3LYP/6-31+G(d,p) and M06-2X/6-311++G(d,p). Results showed that the two reaction routes had very similar trends energetically, the difference between the energy levels of the corresponding molecules was just 6.13 kJ/mol on average. The most stable structure was obtained in the benzo(a)anthracene pathway.

## 1. Introduction

**Figure 1:**
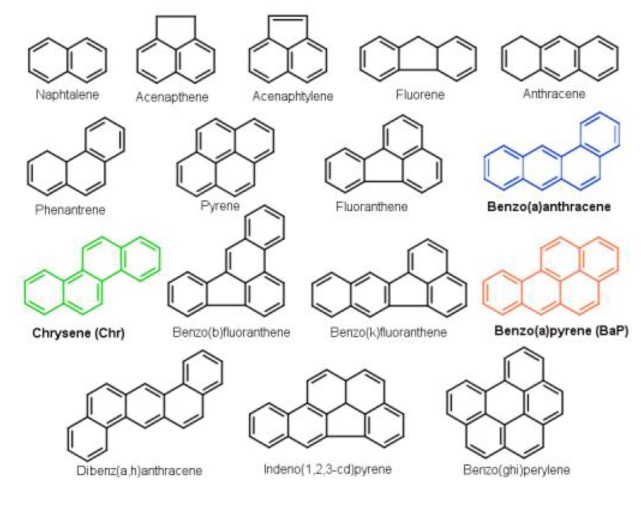
Chemical structure of the “parent PAH” molecules. The colored molecules (Benzo(a)anthracene, Chrysene and Benzo(a)pyrene) are involved in the studied reaction mechanism.

Polycyclic aromatic hydrocarbons (PAHs) consist of a set of several thousands of compounds of ubiquitous pollutants in the environment. They have structures composed of multiple aromatic rings creating a concern to people’s health [1,2]. The intensity of their monitoring in the environment started more than 40 years ago, with the appearance of a list issued by the U.S. Environmental Protection Agency (EPA) in 1976 [3,4]. That list contained 16 priority PAHs (often called “parent PAHs”, Figure 1). Up until now, the parent PAH molecules have been regarded by researchers as being representatives for all the PAHs. However, some recent studies strongly suggest further completion of the list in order to cover the wide range of polycyclic aromatic components that occur in any studied samples [5,6,7]. The priority PAHs have also been included in the Convention of Long-range Transboundary Air Pollution Protocol on Persistent Organic Pollutants [8].

Regarding their origin, PAHs can be released into the atmosphere due to natural processes (wild fires, volcano eruptions, erosion of ancient sediment etc.) but the vast majority of their emission is related to anthropogenic activities [9].

In both natural and man-made liberation processes the formation of PAH molecules is caused by the incomplete combustion of carbonaceous material, through pyrolysis and pyrosynthesis processes [10].

Although the global emission of the parent PAHs shows a slightly descending trend, nevertheless their emission quantity is still too large [11]. The highest priority PAH emissions come from developing countries, however the emissions in developed regions are significant as well [11]. Because of the different energy technology systems of these countries, the dominant PAH emisson sources are also different. Over 80% of the emission has been attributed to developing countries and more than half has originated from biomass and coal burning [11].

The adverse effect of the PAH molecules is also highlighted by the fact that they are considered as soot precursors [12,13]. Of course soot formation also reduces the combustion efficiency [14].

In order to prevent or reduce the production of these compounds, a thorough understanding of their formation and growth mechanisms is required. An anticipating combustion chemistry model for PAH formation is based on the description of the formation of the “first aromatic ring” and an additional explanation of the molecular growth reactions [15]. Basically, benzene was considered as being the first aromatic ring in PAH formation [16,17], however some reactions have proven that aromatics can form without the presence of benzene as well [16,18,19]. A suitable example for this is the published work by Cavalotti and Polino [20] in which the formation of naphtalane with two cyclopentadienyl radicals (^●^C_5_H_5_) was described.

A well known alternative PAH growth reaction is the hydrogen abstraction and acetylene (C_2_H_2_) addition (HACA) mechanism [21]. However, some subsequent control experiments have demonstrated that the HACA mechanism underpredicts the concentrations of PAHs in flames [22] and is too slow to account for the fast process of PAH formation [23,24]. The study of Kislov and co-workers [25] also serves as compelling evidence for the uncertainty of the HACA. They intented to show computationally that HACA could grow additional rings on aromatic hydrocarbons, but it was reported that even in the best case only 6% of the total yield could be assigned to six-membered ring growth by C_2_H_2_ addition, and mostly five-membered ring formation had taken place by HACA.

In order to overcome these limitations, improvements for the HACA mechanism and other new reaction pathways for PAH formation were suggested. For instance benzene and naphtalene formation was studied via numerous resonantly stabilized radicals such as vinyl [26], propargyl [27,28,29], cyclopentadienyl [22,30,31,32]. A combination of PAH condensation and the HACA mechanism was also proposed [22], within which a phenyl ring addition took place and then C_2_H_2_ was added to form two aromatic rings.

It is important to mention the famous Diels–Alder mechanism as well [33,34], where C_2_H_2_ addition takes place, which is followed by the loss of a H_2_. Nonetheless, with computational chemistry studies it was shown that the Diels–Alder reaction was slower than the HACA mechanism [35].

Shukla et al. [36] suggested the phenyl-addition-cyclization (PAC) mechanism because in their experiments numerous PAHs with a mass difference of 75 amu were observed. In this mechanism a phenyl radical was added to a PAH, which was followed by hydrogen abstraction desorption, and finally within the loss of a hydrogen molecule, a ring formation took place.

Two years later [37] the extensions of PAC routes with the HACA mechanism was suggested, because none of them alone seemed to be good enough to compete with the speed of soot formation, due to their different dependences on the site preference of the starting species.

It is essential to note the importance of alkyl radicals in the formation of polycyclic aromatic hydrocarbons from fuels with alkylaromatic compounds. Methyl radical is one of the major products generated from aromatic fuels, which plays a crucial role in the formation of C_3_H_3_ radicals, which is the basic step for benzene formation. Therefore, not surprisingly another mechanism for PAH growth, namely the methyl addition/cyclization (MAC), which involves the addition of two or three methyl radicals on PAHs has emerged. This mechanism could lead to the formation of a new ring in aromatic structures via hydrogen loss. In the experiment conducted by Shukla et al., on toluene pyrolisis, PAH mass spectra were obtained, which were separated by 14 amu. This had clearly revealed the presence of methyl radicals (^●^CH_3_) in PAH formation [38].

Altogether, based on the above described mechanisms, it can be stated that in the last few decades the examinations of the molecular growth of small precursor molecules to large PAHs has become one of the central subjects in combustion chemistry [39,40]. The resultant numerous concepts were based mostly on experimental studies [14,29,41,42,43]. Although, because of the complexity and diversity of the molecules the examinations are far from being over. In such situations quantum chemical calculations can be succesfully applied as a solution in order to elucidate the thermodynamics and kinetics of the formation of PAHs [44,45]. Taking these into consideration, in the present work, two new formation pathways of benzo(a)pyrene (BaP) were proposed starting from chrysene (Chr) or benzo(a)anthracene (BaA). BaP, Chr and BaA are members of the 16 priority PAHs (Figure 1). The carcinogenic risks of these molecules were emphasised by the International Agency for Research on Cancer classifying benzo(a)pyrene in the first class and the reactants in the B2 class [46]. Therefore, it is important to understand their reactions and formations. The studied BaA → BaP and Chr → BaP reaction pathways were based on the aforementioned MAC mechanism and their energetic features were successfully investigated and compared.

## 2. Computational Methods

In order to determine the energy level of the reactants, intermediates, transition states and products that occured in the developed reaction mechanisms for BaP formation, the Gaussian 09 program package was used [47]. For geometry optimisations, two density functional theory (DFT) methods were applied, the Becke three parameter hybrid method with Lee–Yang–Parr correlation functional approximation (B3LYP) for preliminary calculations [48] and the highly effective Minnesota hybrid meta exchange correlation functional (M06-2X) [49]. B3LYP was used in combination with a valence double-ζ basis set including diffuse and polarization functions on heavy atoms and polarization functions for hydrogen (6-31+G(d,p)), while M06-2X calculations were carried out by using the 6-311++G(d,p) basis set [50,51]. The structures were pre-optimized at the B3LYP/6-31+G(d,p) level of theory. Then, the M06-2X calculations were performed with the default (99 radial shells and 590 angular points per shell) and a finetuned integration grid (99 radial shells and 974 angular points per shell) as well.

Both functionals proved to be successful in the study of various aspects of PAHs [29,52]. However, it was revealed recently, that M06-2X in some cases underestimates activation Gibbs free energies [53]. Therefore, activation Gibbs free energy values obtained by using the two functionals were compared for verification purposes.

**Figure 2:**
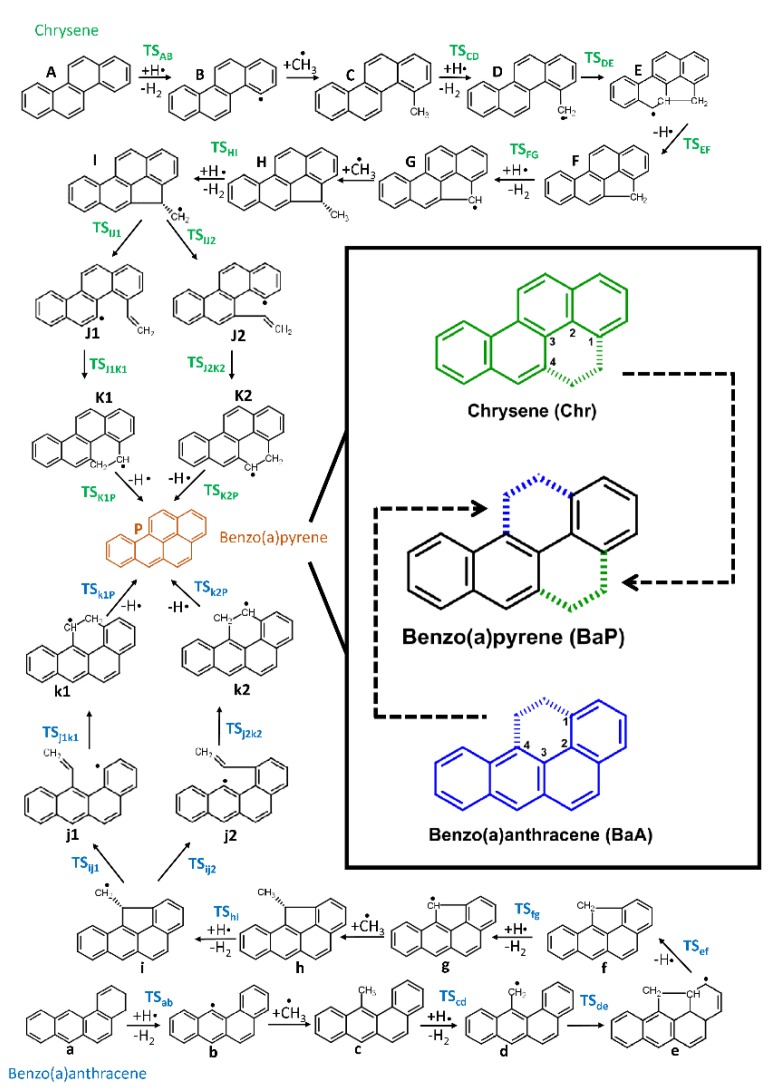
Reaction pathways of the formation of benzo(a)pyrene (”**P**”) starting from chrysene (”**A**”) or benzo(a)anthracene (”**a**”). 2D structures of chrysene, benzo(a)pyrene, benzo(a)anthracene are highlighted along with the carbon atoms, which were involved in the new ring formations.

Critical stationary points were characterized by frequency calculations at both level of theories. In addition, to verify that the computed transition states (first-order saddle points) actually connected the desired starting materials and products (local minima). Intrinsic reaction coordinate (IRC) analysis [54] was carried out at the M06-2X/6-311++G(d,p) level of theory. The reaction and activation Gibbs free energies as well as the relative energies of the studied molecules were calculated. The reference levels of the two benzo(a)pyrene formation reaction channels (Figure 2) corresponded to chrysene (Chr) and benzo(a)anthracene (BaA) with four additional hydrogen atoms (4H^●^) and two methyl radicals (2^●^CH_3_) which served as reaction partners. Atomic balance was considered and the number of carbon and hydrogen atoms were kept the same by using additional species (hydrogen atoms, hydrogen molecules and methyl radicals) in each step of benzo(a)pyrene formation (Figure S2).

## 3. Results and Discussion

In this work two MAC mechanism-based BaP formation pathways were investigated starting either with Chr or BaA (Figure 2). Both reaction routes included 25 structures: 13 intermediate structures and 12 transition state structures. The intermediate species found in the reaction pathways are denoted alphabetically with lower- and upper case letters (Benzo(a)anthracene = ”**a**”; Chrysene = ”**A**”, Figure 2). The transition state structures between successive intermediates such as **A** and **B** are denoted as a **TS** having **A** and **B** together in subscript (**TS_AB_**).

**Figure 3:**
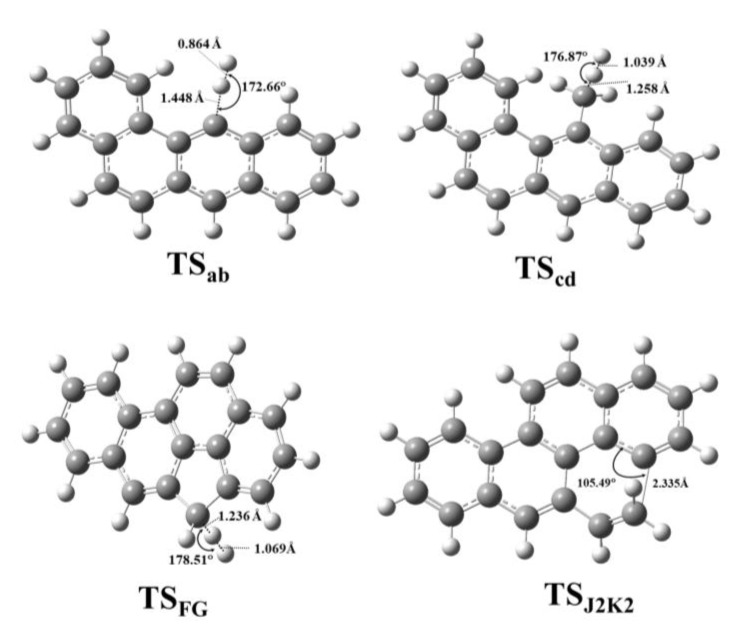
Representative transition state structures of benzo(a)pyrene formation starting from benzo(a)anthracene (**TS_ab_** and **TS_cd_**) and chrysene (**TS_FG_** and **TS_J2K2_**) were located at the M06-2X/6-311++G(d,p) level of theory using a finetuned integration grid (99 radial shells and 974 angular points per shell) and depicted along with interatomic distances and bond angle.

The obtained geometric parameters were well in line with the experimental values [55,56], having a relative error less then 2% in average for chrysene for both levels of theories (Tables S1 and S2). However, in the case of benzo(a)pyrene the relative error were 0.5% and 4.1% on average for M06-2X and B3LYP functionals, respectively. The structural parameters of the transition states such as the bond lengths and angles were collected (Table S3, Figures S3–S6). Representative transition state structures (**TS_ab_**, **TS_cd_**, **TS_FG_**, **TS_J2K2_**) along with the geometrical parameters are depicted in Figure 3.

Similarities among the structures belonging to the same reaction types (i.e., hydrogen abstractions, ring formation etc.) could be observed. Comparing the two reaction routes the difference between the corresponding bond angles and bond lengths was up to 1.08° and 0.012 Å, respectively. However, this was still small enough to prove that the selected methods were appropriate to handle and describe the geometrical features of the studied species.

To further verify the method selection, homolytic bond dissociation energies (BDE) of naphthalane, which is the simplest PAH, have been computed and the calculated values were compared to the experimental data [57]. The calculated and experimentally measured BDEs were in good agreement to each other as the deviation (BDE_exp_ - BDE_calc_) was only 2.7 kJ/mol and 3.8 kJ/mol for B3LYP/6-31+G(d,p) and M06-2X/6-311++G(d,p), respectively. Thus, the selected model chemistries were reliable and could be used to compute thermochemical parameters of polycyclic aromatic hydrocarbons.

The computed relative thermodynamic functions (Δ*G*, Δ*H*), which correspond to the structures involved in benzo(a)pyrene formation (Figure 2) starting from benzo(a)anthracene or chrysene are summarized in Table 1 and Table S4. The endergonicities and exergonicities of the processes are clearly seen in these tables. The activation Gibbs free energy values of the reactions were calculated and are enumerated in Tables S7 and S8. In the case of the chrysene route the Gibbs free energy of activation values obtained with B3LYP/6-31+G(d,p) were lower (13.0 kJ/mol on average) than those obtained with M06-2X/6-311++G(d,p) in all cases (Table S8). This was also the case for the benzo(a)anthracene route, with the exception of TS_ef_ structure (Table S6), which was higher with 2 kJ/mol, than the values obtained with M06-2X/6-311++G(d,p).

**Figure 4:**
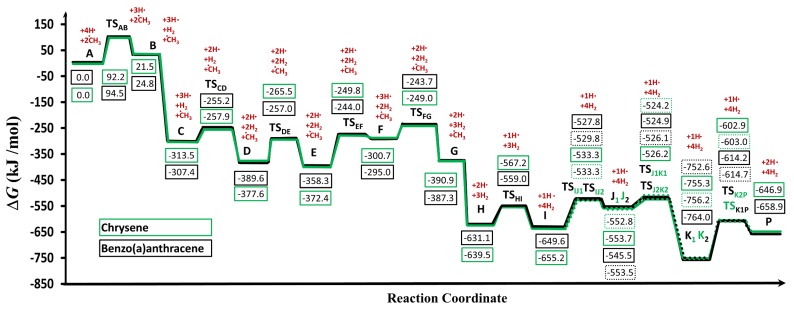
Gibbs free energy profile of the two reaction pathways leading to the formation of benzo(a)pyrene (BaP, **P**) calculated at the M06-2X/6-311++G(d,p) level of theory using a fine-tuned integration grid (99 radial shells and 974 angular points per shell). The two reaction pathways are indicated by green and black (solid /dotted) lines, in the case of chrysene and benzo(a)anthracene, respectively.

In order to evaluate and compare energetically the reaction mechanisms, a potential energy diagram was drawn (Figure 4). It clearly shows, that both reaction routes followed a similar trend. Both of the proposed pathways included the alternation of the two essential steps. These were the hydrogen abstractions to create radical sites on PAH structures, which was followed by the methyl addition. The hydrogen abstraction was achieved by either an additional hydrogen atom or by self cleavage of the carbon-hydrogen bonds, which led to a carbon-centered radical. Then, the addition of a methyl group to the previously formed free radical site could occur.

Based on the structural features of the starting species (”**A**” and ”**a**” structures in Figure 2) the growth mechanisms were started by hydrogen abstractions following the attack of external H-atoms from the first and fourth carbon atoms (Figure 2) in the case of chrysene and benzo(a)anthracene, respectively. These steps resulted in **B** and **b** radical intermediates (Figure 2 and Figure 4) with the following activation Gibss free energy values: Δ*G*^‡^(**TS_AB_**) = 92.2 kJ/mol and Δ*G*^‡^(**TS_ab_**) = 94.5kJ/mol. Furthermore the reaction Gibbs free energies for these endergonic reactions were 21.5 kJ/mol and 24.8 kJ/mol, in the case of **B** and **b**, respectively (Table 1). The reactions were continued with barrierless additions of methyl groups on the free radical sites of **B** and **b** in order to form methyl-chrysene and metyl-benzo(a)anthracene structures (**C** and **c**). These steps represented the most exergonic phases among the reaction mechanism, having Δ*G*(**BC**) = −334.9 kJ/mol and Δ*G*(**bc**) = −332.1 kJ/mol reaction Gibbs free energy values for the Chr and BaA channel, respectively. The reaction routes continued with hydrogen abstractions from the methyl groups of **C** and **c**. These were performed by external H atoms, the activation Gibbs free energies required to obtain **D** and **d** structures were Δ*G*^‡^(**TS_CD_**) = 55.6 kJ/mol and Δ*G*^‡^(**TS_cd_**) = 52.1 kJ/mol. These activation Gibbs free energy values were 1.7 times lower on average, than those required for the **B** and **b** formation from **A** and **a**, due to the difference between the strengths of the C-H bonds in the aromatic structures compared to the aliphatic ones. The reactions were followed by the formations of five-membered ring sturctures (**E** and **e**) through the attack by the methylene groups at the 4^th^ carbon in chrysene and the first carbon atom in BaA (Figure 2). This process occurred after overcoming the neccesary Gibbs free energy of activation (Δ*G*^‡^(**TS_DE_**) = 112.2 kJ/mol and Δ*G*^‡^(**TS_de_**) = 132.6 kJ/mol, for the Chr and BaA route, respectively). In the next step, a hydrogen from the fourth and the first carbon atom has been eliminated in the case of chrysene and benzo(a)anthracene pathways respectively, which led to the formation of the **F** and **f** structures. These endergonic reactions required Δ*G***(EF)** = 71.7 kJ/mol and Δ*G*(**ef**) = 63.3 kJ/mol reaction Gibbs free energies. For the reaction to proceed further, H abstraction from **F** and **f** molecules had taken place, after overcoming a Gibbs free energy of activation of Δ*G*^‡^(**TS_FG_**) = 51.7 kJ/mol and Δ*G*^‡^(**TS_fg_**) = 51.3 kJ/mol, reaching the intermediate radical species **G** and **g** with an energy lower that 90.2 kJ/mol and 92.3 kJ/mol compared to the previous intermediates. At this point of the reaction mechanism, the second barrierless methyl addition occured. As **G** and **g** were planar, this barrierless methyl addition could have occurred from both sides of the plane, but it will have led to energetically equivalent species. Therefore, only one process was considered and will be discussed. This step increased the stability of the resulted **H** and **h** intermediate by 240.3 kJ/mol and 252.2 kJ/mol with respect to the previous structures (**G** and **g**). The formation of the **I** and **i** radical intermediate was attributed to another H abstraction from the methyl group by an external H atom, which required an activation Gibbs free energy of Δ*G*^‡^(**TS_HI_**) = 72.3 kJ/mol and Δ*G*^‡^(**TS_hi_**) = 72.2 kJ/mol. In the next step, as it is shown in Figure 2, the reaction pathways were split into two routes, to obtain **J1** and **J2** as well as **j1** and **j2** structures by ring openings. The reaction routes were continuted by simultaneous bond breakings and bond formations. In the case of **J2** and **j1** the ring opening occured at the first carbon, while in the case of **J1** and **j2** this took place at the 4^th^ carbon. **TS_IJ1_** had the highest Gibbs free energy of activation values among these steps with ΔG^‡^(**TS_IJ2_**) = 121.9 kJ/mol. However, the difference between the corresponding Gibbs free energies (**TS_IJ1_**, **TS_ij1_** and **TS_IJ1_**, **TS_ij1_**) was just 1.16 kJ/mol on average. All **J1**, **J2**, **j1** and **j2** structures were formed by endothermic reactions. The fifth six-membered ring was formed in **K1** and **k1** as well as **K2** and **k2** structures, with a 205.7 kJ/mol reaction Gibbs free energy value on average. The most stable structure among the four resulting molecules was k1 with −764.0 kJ/mol, after overcoming an Δ*G*^‡^(**TS_j1k1_**) = 20.6 kJ/mol Gibbs free energy of activation. The highest activation Gibbs free energy values appeared on the last step for both routes, with Δ*G*^‡^(**TS_K2P_**) = 153.3 kJ/mol and Δ*G*^‡^(**TS_k1P_**) = 149.0 kJ/mol for chrysene and benzo(a)anthracene, respectively. As it can be seen, between the two routes of this part of the reaction (**K1** and **K2**; **k1** and **k2**; Figure 2), the left-hand side of the benzo(a)anthracene pathway was more preferable energetically than the right-hand side (**k1** < **k2**). However, in the chrysene pathway the opposite occured, obtaining on the right hand side a more stable molecule (**J2** < **J1**). The penultimate steps were again H eliminations, through which the final product, benzo(a)pyrene was achieved, with a stability of −646.9 kJ/mol and −658.9 kJ/mol, respectively.

## 4. Conclusions

The formation of the strongly carcinogenic benzo(a)pyrene was studied by applying a newly developed methyl addition/cyclization (MAC) mechanism. Two reaction pathways were proposed starting from chrysene and benzo(a)anthracene (consisting of four aromatic rings), respectively. The reaction routes had the same reaction steps, both included four hydrogen abstractions, two methyl radical additions, three hydrogen atom eliminations, one ring closure and one rearrangement. Energetically the first methyl additions were the most exergonic steps, placing the resultant molecules to –313.5 kJ/mol and –307.4 kJ/mol (**C**, **c**) in case of the chrysene and benzo(a)anthracene, respectively. The two reaction pathways had very similar trends energetically, the difference between the energy levels of the corresponding molecules was just 6.13 kJ/mol on average. The **k1** structure in the benzo(a)anthracene → benzo(a)pyrene pathway became the most stable molecule with Δ*G* = −764.0 kJ/mol. All in all, the formation of benzo(a)pyrene from chrysene and benzo(a)anthracene could be reached with this newly proposed MAC mechanism.

## Figures and Tables

**Table 1:** Relative Gibbs free energy (Δ*G,* kJ/mol*),* relative enthalpy (Δ*H,* kJ/mol), and enthropy (*S*, cal/mol*K) values of benzo(a)pyrene (BaP) formation mechanism starting from chrysene (Chr→BaP) or benzo(a)anthracene (BaA→BaP) computed at the M06-2X/6-311++G(d,p) level of theory using a fine-tuned integration grid (99 radial shells and 974 angular points per shell), at 298.15 K and 1 atm within the harmonic oscillator rigid rotor approximation.

BaA→BaP	Δ*G*	Δ*H*	*S*	Chr→BaP	Δ*G*	Δ*H*	*S*
**a**	0.00	0.00	108.46	**A**	0.00	0.00	108.91
**TS_ab_**	94.50	67.86	114.51	**TS_AB_**	92.24	64.50	114.06
**b**	24.76	30.63	109.44	**B**	21.46	26.89	109.54
**c**	−307.39	−359.74	113.13	**C**	−313.47	−364.87	114.34
**TS_cd_**	−255.24	−336.50	117.34	**TS_CD_**	−257.86	−339.06	117.84
**d**	−389.58	−435.92	114.22	**D**	−377.64	−424.03	114.62
**TS_de_**	−256.98	−306.65	111.54	**TS_DE_**	−265.48	−315.72	111.53
**e**	−358.30	−408.32	111.27	**E**	−372.38	−422.91	111.31
**TS_ef_**	−244.05	−294.14	111.21	**TS_EF_**	−249.81	−300.47	111.21
**f**	−295.00	−314.47	108.36	**F**	−300.71	−320.71	108.38
**TS_fg_**	−243.72	−291.55	113.02	**TS_FG_**	−248.99	−297.58	112.86
**g**	−387.31	−401.11	109.19	**G**	−390.87	−405.51	108.96
**h**	−631.14	−700.50	115.01	**H**	−639.50	−708.80	115.49
**TS_hi_**	−558.99	−656.73	119.65	**TS_HI_**	−567.16	−664.70	120.25
**i**	−649.59	−710.62	117.95	**I**	−655.24	−715.79	118.78
**TS_ij1_**	−527.83	−590.35	117.05	**TS_IJ1_**	−533.35	−595.99	117.10
**TS_ij2_**	−529.88	−592.03	116.76	**TS_IJ2_**	−533.31	−596.02	117.05
**j_1_**	−545.54	−603.31	120.90	**J_1_**	−553.65	−611.28	121.12
**j_2_**	−553.48	−610.83	120.57	**J_2_**	−552.85	−610.59	121.03
**TS_j1k1_**	−524.89	−587.64	116.58	**TS_J1K1_**	−526.23	−588.62	117.31
**TS_j2k2_**	−526.08	−588.47	116.87	**TS_J2K2_**	−524.15	−586.46	117.38
**k_1_**	−764.02	−827.12	116.29	**K_1_**	−755.42	−818.33	116.88
**k_2_**	−752.55	−818.11	114.32	**K_2_**	−756.29	−820.14	116.13
**TS_k1P_**	−614.19	−678.89	115.01	**TS_K1P_**	−602.87	−668.16	114.99
**TS_k2P_**	−614.69	−679.93	114.58	**TS_K2P_**	−602.96	−668.26	114.97
**P**	−658.89	−694.43	110.99	**P**	−646.93	−683.02	110.99

## Supplementary Materials

The following are available online, validation of the applied methods and comparison of the structural parameters (bond lengths, angles) of the transition state structures. The relative Gibbs free energy*,* relative enthalpy, enthropy values and activation Gibbs free energy values computed at the B3LYP/6-31+G(d,p) and M06-2X/6-311++G(d,p) levels of theory and their comparison. The transition state structures along with the interatomic distances and bond angles at the given reaction site.
