# Supplementary material for: Formation Mechanism of Benzo(a)pyrene: One of the Most Carcinogenic Polycyclic Aromatic Hydrocarbons (PAH)
Source: Molecules. 2019 Mar 15;24(6):1040. doi: 10.3390/molecules24061040 (PMC6470522; doi:10.3390/molecules24061040)
Supplement: Supplementary file 1 [file molecules-24-01040-s001.zip › Reizer-etal-2019-Supplementary-rev2.docx]

**Formation Mechanism of Benzo(a)pyrene one of the Most Carcinogenic Polycyclic Aromatic Hydrocarbon (PAH)**

Edina Reizer^1^, Imre G. Csizmadia^1,2^, Árpád B. Palotás^3^, Béla Viskolcz^1^, Béla Fiser^1,4^

*^1^Institute of Chemistry, University of Miskolc, Miskolc-University town, Hungary 3515*

*^2^Department of Chemisrty, University of Toronto, Ontario, Canada*

*^3^Institute of Energy and Quality Affairs, University of Miskolc, Miskolc-University town, Hungary3515*

*^4^Ferenc Rákóczi II. Transcarpathian Hungarian Institute, Beregszász, Transcarpathia, 90200-Ukraine*

*E-mail:* [fiser.bela@gmail.com](mailto:fiser.bela@gmail.com)

**SUPPLEMENTARY INFORMATION**

| **Table S1** The experimental and computed bond lengths values of chrysene and the relative errors for both level of theories | | | | | |
| --- | --- | --- | --- | --- | --- |
| **Bonds in chrysene** | **Experimental values^1^**  **(Å)** | **Calc**  **M06-2X/6-311++G(d,p)**  **(Å)** | **Relative error**  **(%)** | **Calc**  **B3LYP/6-31+G(d,p)**  **(Å)** | **Relative error**  **(%)** |
| C1-C2  C2-C3  C3-C4  C4-C5  C5-C6  C6-C7  C8-C9  C9-C10  C10-C1  C2-C7 | 1.409  1.433  1.423  1.387  1.392  1.435  1.410  1.403  1.402  1.390 | 1.450  1.415  1.375  1.407  1.372  1.413  1.426  1.356  1.405  1.415 | 2.9  1.6  3.7  0.9  1.6  1.7  0.7  3.8  0.2  1.4 | 1.454  1.419  1.382  1.410  1.380  1.417  1.427  1.365  1.418  1.428 | 2.9  1.6  2.9  1.7  0.9  1.3  1.2  2.7  1.1  2.7 |

| **Table S2** The experimental and computed bond lengths values of benzo(a)pyrene and the relative errors for both level of theories. | | | | | |
| --- | --- | --- | --- | --- | --- |
| **Bonds in benzo(a)pyrene** | **Experimental values**  **(Å)** | **Calc**  **M06-2X/6-311++G(d,p)**  **(Å)** | **Relative error**  **(%)** | **Calc**  **B3LYP/6-31+G(d,p)**  **(Å)** | **Relative error**  **(%)** |
| C1-C2  C2-C3  C3-C4  C4-C5  C5-C6  C6-C7  C7-C8  C8-C9  C9-C10  C10-C11  C11-C12  C12-C13  C13-C14  C14-C15  C15-C16  C16-C17  C17-C18  C18-C19  C19-C20  C1-C18  C3-C20  C7-C20  C10-C19  C12-C17 | 1.352  1.441  1.412  1.375  1.378  1.402  1.433  1.342  1.446  1.361  1.418  1.425  1.374  1.397  1.364  1.418  1.436  1.393  1.419  1.423  1.417  1.415  1.444  1.410 | 1.357  1.429  1.405  1.381  1.396  1.391  1.445  1.347  1.446  1.374  1.413  1.420  1.367  1.412  1.370  1.419  1.438  1.407  1.429  1.432  1.416  1.422  1.436  1.422 | 0.4  0.8  0.5  0.4  1.3  0.8  0.8  0.0  0.0  1.0  0.4  0.4  0.5  1.1  0.4  0.1  0.1  1.0  0.7  0.6  0.1  0.5  0.6  1.6 | 1.433  1.367  1.430  1.411  1.389  1.401  1.400  1.443  1.358  1.443  1.385  1.416  1.424  1.376  1.416  1.416  1.422  1.444  1.432  1.433  1.427  1.431  1.443  1.435 | 6.0  5.0  1.3  2.6  0.8  0.1  2.3  7.5  6.1  6.1  2.3  0.7  3.7  1.5  3.8  0.2  1.0  3.6  0.9  0.7  0.7  1.1  0.1  2.5 |

| 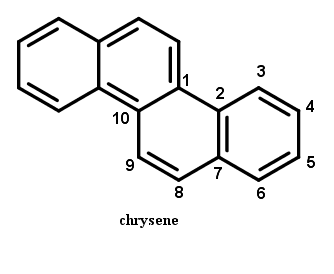 | 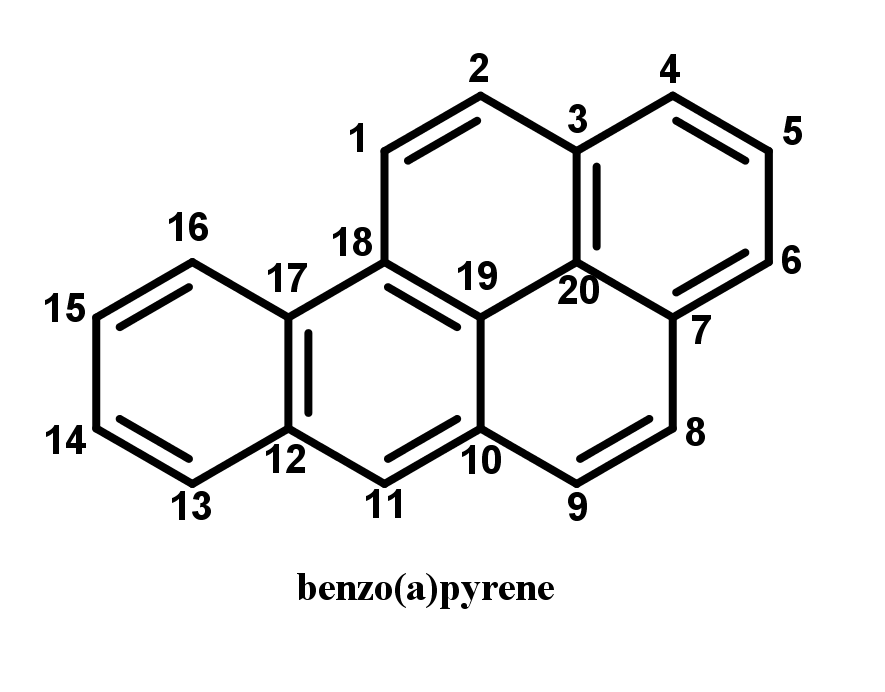 |
| --- | --- |
| **Figure S1** Atom labeling scheme used in Table S1 and S2 for chrysene and benzo(a)pyrene respectively | |

**Table S3** Structural parameters (bond lengths, angles) of transition state structures of benzo(a)pyrene (BaP) formation reaction mechanisms starting from benzo(a)anthracene (BaA→BaP) or chrysene (Chr→BaP). The structures were computed at the M06-2X/6-311++G(d,p) level of theory with finetuned integration grid (99 radial shells and 974 angular points per shell), at 298.15 K and 1 atm within the harmonic oscillator rigid rotor approximation.

| **Reaction Type** | **Transition State** | **Angles** | | | **Bond length (****Å)** | | | | | |
| --- | --- | --- | --- | --- | --- | --- | --- | --- | --- | --- |
|  |  |  | **BaA → BaP** | **Chr → BaP** | **H-H**  **(BaA→ BaP)** | **H-H**  **(Chr→ BaP)** | **C-H**  **(BaA→ BaP)** | **C-H**  **(Chr→ BaP)** | **C-C**  **(BaA→ BaP)** | **C-C**  **(Chr→ BaP)** |
| **Hydrogen**  **abstraction** | TS_ab_ /TS_AB_ | C-H-H | 172.66° | 169.44° | 0.864 | 0.868 | 1.448 | 1.430 | - | - |
|  | TS_cd_ /TS_CD_ | C-H-H | 176.87° | 176.73° | 1.039 | 1.017 | 1.258 | 1.269 | - | - |
|  | TS_fg_ /TS_FG_ | C-H-H | 177.87° | 178.52° | 1.069 | 1.069 | 1.237 | 1.236 | - | - |
|  | TS_hi_ /TS_HI_ | C-H-H | 172.32° | 171.84° | 0.911 | 0.910 | 1.376 | 1.378 | - | - |
| **Hydrogen**  **dissociation** | TS_ef_ /TS_EF_ | C-C-H | 92.90° | 97.72° | - | - | 1.788 | 1.802 | - | - |
|  | TS_k1P_ /TS_K1P_ | H-C-H | 84.24° | 84.68° | - | - | 1.892 | 1.906 | - | - |
|  | TS_k2P_ /TS_K2P_ | H-C-H | 82.43° | 84.12° | - | - | 1.878 | 1.907 | - | - |
|  | TS_de_ /TS_DE_ | C-C-C | 91.37° | 91.04° | - | - | - | - | 2.135 | 2.141 |
| **Ring Opening/ Ring**  **Closure** | TS_ij1_ /TS_IJ1_ | C-C-C | 107.98° | 110.41° | - | - | - | - | 2.954 | 2.997 |
|  | TS_ij2_ /T_SIJ2_ | C-C-C | 110.42° | 110.70° | - | - | - | - | 2.990 | 2.999 |
|  | TS_j1k1_ /T_J1K1_ | C-C-C | 105.72° | 105.62° |  |  |  |  | 2.356 | 2.334 |
|  | TS_j2k2_ /T_J2K2_ | C-C-C | 106.00° | 105.49° |  |  |  |  | 2.342 | 2.335 |


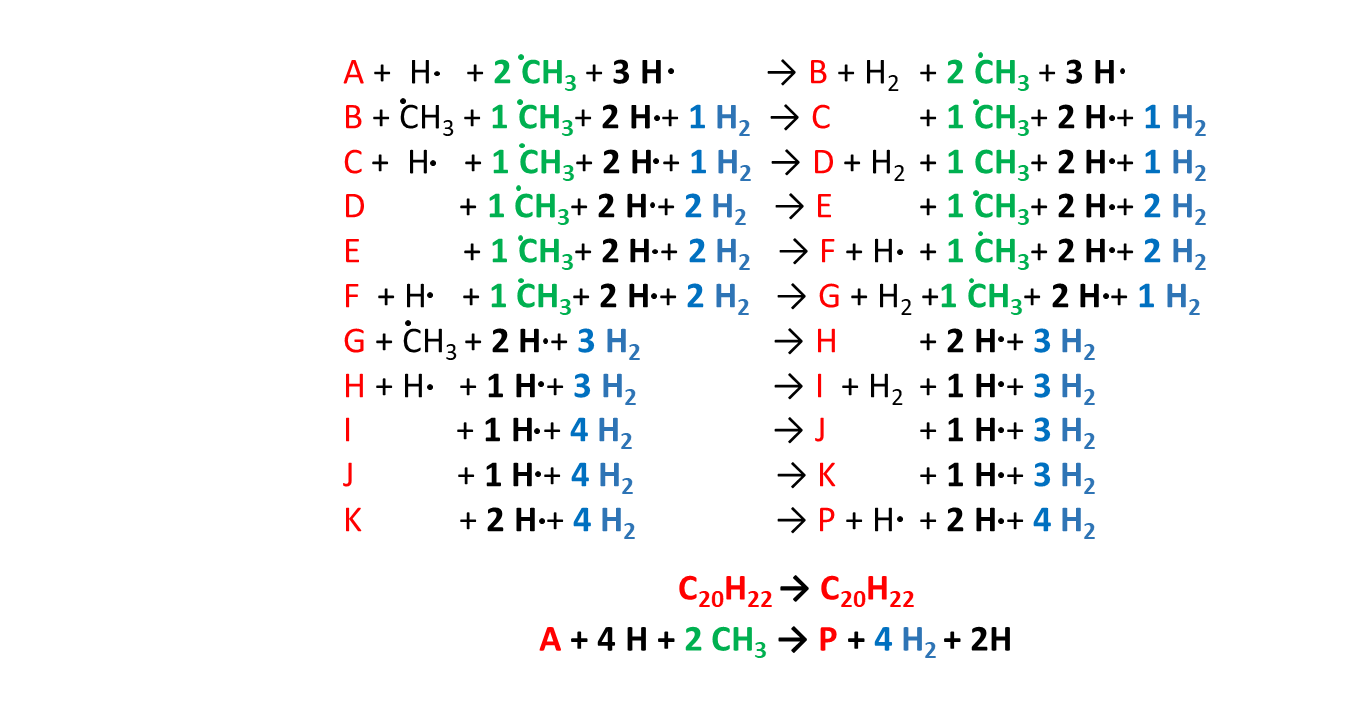


**Figure S2** Atomic balance considered and the number of carbon and hydrogen atoms kept the same by using additional species (hydrogen atoms, hydrogen molecules and methyl radicals) in each step of benzo(a)pyrene formation.

**Table S4** Relative Gibbs free energy (Δ*G*, kJ/mol)*,* relative enthalpy (Δ*H*, kJ/mol), and enthropy (*S,* cal/mol*K) values of benzo(a)pyrene (BaP) formation starting from chrysene (Chr→BaP) or benzo(a)anthracene (BaA→BaP) computed at the B3LYP/6-31+G(d,p) level of theory, at 298.15 K and 1 atm within the harmonic oscillator rigid rotor approximation.

| **BaA→BaP** | **Δ*G*** | **Δ*H*** | ***S*** | **Chr→BaP** | **Δ*G*** | **Δ*H*** | ***S*** |
| --- | --- | --- | --- | --- | --- | --- | --- |
| a | 0.00 | 0.00 | 108.18 | A | 0.00 | 0.00 | 108.82 |
| TS_ab_ | 71.09 | 44.11 | 113.94 | TS_AB_ | 68.21 | 40.25 | 113.80 |
| b | 9.51 | 15.69 | 109.40 | B | 7.47 | 13.17 | 109.65 |
| c | -290.51 | -338.63 | 113.75 | C | -300.87 | -348.31 | 114.94 |
| TS_cd_ | -263.82 | -341.12 | 117.75 | TS_CD_ | -270.88 | -348.33 | 118.27 |
| d | -398.29 | -440.81 | 114.49 | D | -390.60 | -433.42 | 114.90 |
| TS_de_ | -269.80 | -315.93 | 111.61 | TS_DE_ | -279.91 | -326.88 | 111.57 |
| e | -359.81 | -406.52 | 111.14 | E | -372.40 | -419.58 | 111.40 |
| TS_ef_ | -243.55 | -289.96 | 111.37 | TS_EF_ | -250.92 | -298.16 | 111.35 |
| f | -285.31 | -301.66 | 108.09 | F | -292.77 | -309.93 | 108.07 |
| TS_fg_ | -257.64 | -302.44 | 112.67 | TS_FG_ | -265.13 | -310.82 | 112.60 |
| g | -406.68 | -417.38 | 108.87 | G | -412.49 | -424.25 | 108.66 |
| h | -607.94 | -670.78 | 114.96 | H | -619.12 | -682.44 | 115.21 |
| TS_hi_ | -559.55 | -650.43 | 119.87 | TS_HI_ | -570.69 | -662.01 | 120.16 |
| i | -642.93 | -697.18 | 118.10 | I | -652.74 | -706.71 | 118.97 |
| TS_ij1_ | -538.37 | -594.71 | 116.42 | TS_IJ1_ | -547.10 | -604.03 | 116.60 |
| TS_ij2_ | -543.29 | -599.40 | 116.61 | TS_IJ2_ | -547.10 | -603.93 | 116.68 |
| j_1_ | -545.48 | -596.52 | 120.67 | J_1_ | -557.01 | -608.03 | 121.33 |
| j_2_ | -557.09 | -607.36 | 121.29 | J_2_ | -556.09 | -607.25 | 121.23 |
| TS_j1k1_ | -531.35 | -586.90 | 117.05 | TS_J1K1_ | -536.28 | -592.14 | 117.46 |
| TS_j2k2_ | -535.84 | -591.36 | 117.08 | TS_J2K2_ | -534.48 | -590.19 | 117.58 |
| k_1_ | -765.90 | -823.38 | 115.51 | K_1_ | -759.77 | -817.89 | 115.64 |
| k_2_ | -754.66 | -813.79 | 114.19 | K_2_ | -760.77 | -819.21 | 115.39 |
| TS_k1P_ | -620.50 | -678.35 | 115.22 | TS_K1P_ | -612.29 | -670.99 | 115.18 |
| TS_k2P_ | -619.61 | -677.71 | 115.02 | TS_K2P_ | -612.39 | -671.12 | 115.16 |
| P | -652.42 | -681.82 | 110.62 | P | -644.03 | -674.24 | 110.62 |

**Table S5** Relative Gibbs free energy values (Δ*G*, kJ/mol) of benzo(a)pyrene (BaP) formation mechanism starting from chrysene (Chr → BaP) computed at the B3LYP/6-31+G(d,p) [B3LYP] and M06-2X/6-311++G(d,p) [M06-2X] levels of theory. In the latter case, the calculations were performed besides the default integration grid (99 radial shells and 590 angular points per shell) with a finetuned setting as well (99 radial shells and 974 angular points per shell) [M06-2X_gr]. The difference between the relative Gibbs free energies (ΔΔ*G,* kJ/mol) of [M06-2X vs B3LYP] and [M06-2X_gr vs B3LYP] is also tabulated.

| **Chrysene** | **M06-2X** | **M06-2X_gr** | **B3LYP** | **M06-2X  vs  B3LYP** | **M06-2X­_gr  vs  B3LYP** |
| --- | --- | --- | --- | --- | --- |
|  | Δ*G* | | | ΔΔ*G* | |
|  | kJ/mol | | | | |
| A | 0.00 | 0.00 | 0.00 | 0.00 | 0.00 |
| TS_AB_ | 90.71 | 92.24 | 68.21 | 22.49 | 24.03 |
| B | 21.86 | 21.46 | 7.47 | 14.39 | 13.99 |
| C | -314.26 | -313.47 | -300.87 | 13.38 | 12.60 |
| TS_CD_ | -258.50 | -257.86 | -270.88 | 12.38 | 13.02 |
| D | -377.92 | -377.64 | -390.60 | 12.68 | 12.97 |
| TS_DE_ | -262.85 | -265.48 | -279.91 | 17.06 | 14.43 |
| E | -372.31 | -372.38 | -372.40 | 0.09 | 0.02 |
| TS_EF_ | -248.85 | -249.81 | -250.92 | 2.07 | 1.10 |
| F | -299.78 | -300.71 | -292.77 | 7.01 | 7.94 |
| TS_FG_ | -247.90 | -248.99 | -265.13 | 17.23 | 16.13 |
| G | -390.01 | -390.87 | -412.49 | 22.47 | 21.62 |
| H | -638.20 | -639.50 | -619.12 | 19.08 | 20.38 |
| TS_HI_ | -565.19 | -567.16 | -570.69 | 5.50 | 3.53 |
| I | -653.05 | -655.24 | -652.74 | 0.31 | 2.50 |
| TS_IJ1_ | -532.35 | -533.31 | -547.10 | 14.75 | 13.79 |
| TS_IJ2_ | -532.64 | -533.31 | -547.10 | 14.47 | 13.79 |
| J_1_ | -552.64 | -552.85 | -557.01 | 4.37 | 4.16 |
| J_2_ | -552.18 | -553.65 | -556.09 | 3.91 | 2.44 |
| TS_J1K1_ | -525.60 | -526.23 | -536.28 | 10.69 | 10.05 |
| TS_J2K2_ | -523.40 | -524.15 | -228.14 | 11.09 | 10.33 |
| K_1_ | -754.36 | -755.42 | -759.77 | 5.41 | 4.35 |
| K_2_ | -755.36 | -756.29 | -760.77 | 5.42 | 4.49 |
| TS_K1P_ | -602.15 | -602.87 | -612.29 | 10.14 | 9.42 |
| TS_K2P_ | -601.90 | -602.96 | -612.39 | 10.49 | 9.43 |
| P | -646.20 | -646.93 | -644.03 | 2.17 | 2.90 |
| **Average** |  |  |  | 10.0 (10.4*) | 9.5 (9.9*) |

* - reference point (0.0) excluded

**Table S6** Relative Gibbs free energy values (Δ*G*, kJ/mol) of benzo(a)pyrene (BaP) formation mechanism starting from benzo(a)anthracene (BaA → BaP) computed at the B3LYP/6-31+G(d,p) [B3LYP] and M06-2X/6-311++G(d,p) [M06-2X] levels of theory. In the latter case, the calculations were performed besides the default integration grid (99 radial shells and 590 angular points per shell) with a finetuned setting as well (99 radial shells and 974 angular points per shell) [M06-2X_gr]. The difference between the relative Gibbs free energies (ΔΔ*G,* kJ/mol) of [M06-2X vs B3LYP] and [M06-2X_gr vs B3LYP] is also tabulated.

| **BaA** | **M06-2X** | **M06-2X_gr** | **B3LYP** | **M06-2X  vs  B3LYP** | **M06-2X­_gr  vs  B3LYP** |
| --- | --- | --- | --- | --- | --- |
|  | Δ*G* | | | ΔΔ*G* | |
|  | kJ/mol | | | | |
| a | 0.00 | 0.00 | 0.00 | 0.00 | 0.00 |
| TS_ab_ | 94.43 | 94.50 | 71.09 | 23.34 | 23.40 |
| b | 24.67 | 24.76 | 9.51 | 15.16 | 15.25 |
| c | -307.67 | -307.39 | -290.51 | 17.17 | 16.88 |
| TS_cd_ | -254.84 | -255.24 | -263.82 | 8.98 | 8.58 |
| d | -389.35 | -389.58 | -398.29 | 8.94 | 8.70 |
| TS_de_ | -256.42 | -256.98 | -269.80 | 13.38 | 12.83 |
| e | -357.08 | -358.30 | -359.81 | 2.73 | 1.51 |
| TS_ef_ | -242.97 | -244.05 | -243.55 | 0.57 | 0.50 |
| f | -294.18 | -295.00 | -285.31 | 8.86 | 9.68 |
| TS_fg_ | -242.47 | -243.72 | -257.64 | 15.17 | 13.92 |
| g | -386.32 | -387.31 | -406.68 | 20.36 | 19.37 |
| h | -630.03 | -631.14 | -607.94 | 22.09 | 23.20 |
| TS_hi_ | -557.79 | -558.99 | -559.55 | 1.76 | 0.56 |
| i | -647.50 | -649.59 | -642.93 | 4.57 | 6.66 |
| TS_ij1_ | -528.87 | -529.88 | -538.37 | 9.50 | 8.49 |
| TS_ij2_ | -527.49 | -527.83 | -543.29 | 15.80 | 15.46 |
| j_1_ | -552.60 | -553.48 | -545.48 | 7.12 | 8.01 |
| j_2_ | -545.43 | -545.54 | -557.09 | 11.65 | 11.55 |
| TS_j1k1_ | -524.48 | -524.89 | -531.35 | 6.87 | 6.45 |
| TS_j2k2_ | -525.03 | -526.08 | -535.84 | 10.81 | 9.75 |
| k_1_ | -762.51 | -764.02 | -765.90 | 3.39 | 1.88 |
| k_2_ | -751.41 | -752.55 | -754.66 | 3.25 | 2.11 |
| TS_k1P_ | -613.46 | -614.19 | -620.50 | 7.04 | 6.31 |
| TS_k2P_ | -613.84 | -614.69 | -619.61 | 5.77 | 4.92 |
| P | -657.98 | -658.89 | -652.42 | 5.57 | 6.47 |
| **Average** |  |  |  | 9.1 (9.4*) | 8.7 (9.1*) |

* - reference point (0.0) excluded

| **Table S7** Comparison of the activation energy values (Δ*G*^‡^, kJ/mol) of benzo(a)pyrene (BaP) formation mechanism starting from chrysene (Chr → BaP) computed at the B3LYP/6-31+G(d,p) [B3LYP] and M06-2X/6-311++G(d,p) [M06-2X] levels of theory. In the latter case, the calculations were performed besides the default integration grid (99 radial shells and 590 angular points per shell) with a finetuned setting as well (99 radial shells and 974 angular points per shell) [M06-2X_gr]. The difference between the activation energies (ΔΔ*G*^‡^*,* kJ/mol) of [M06-2X vs B3LYP] and [M06-2X_gr vs B3LYP] is also tabulated. | | | | | |
| --- | --- | --- | --- | --- | --- |
| **Chr → BaP** | **M06-2X** | **B3LYP** | **M06-2X_gr** | **M06-2X**  **vs**  **B3LYP** | **M06-2X_gr**  **vs**  **B3LYP** |
|  | Δ*G*^‡^ | | | ΔΔ*G*^‡^ | |
|  | kJ/mol | | | | |
| A - **TS_AB_** - B | 90.71 | 68.21 | 92.24 | 22.49 | 24.03 |
| C - **TS_CD_** - D | 55.76 | 30.00 | 55.62 | 25.77 | 25.62 |
| D - **TS_DE_** - E | 115.07 | 110.70 | 112.16 | 4.37 | 1.46 |
| E - **TS_EF_** - F | 123.47 | 121.48 | 122.57 | 1.98 | 1.08 |
| F - **TS_FG_** - G | 51.87 | 27.64 | 51.71 | 24.24 | 24.07 |
| H **- TS_HI_ -** I | 73.01 | 48.43 | 72.34 | 24.58 | 23.91 |
| I - **TS_IJ1_** - J1 | 120.41 | 105.63 | 121.93 | 15.06 | 16.29 |
| I - **TS_IJ2_ -** J2 | 120.69 | 105.63 | 121.93 | 15.06 | 16.29 |
| J1 - **TS_J1K1_ -** K1 | 27.04 | 20.72 | 27.42 | 6.32 | 6.70 |
| J2 - **TS_J1K2_** - K2 | 28.93 | 21.61 | 28.70 | 7.32 | 7.09 |
| K1 - **TS_K1P_** - P | 152.21 | 147.48 | 152.55 | 4.73 | 5.07 |
| K2 - **TS_K2P_** - P | 153.46 | 148.38 | 153.32 | 5.08 | 4.94 |

| **Table S8** Comparison of the activation energy values (Δ*G*^‡^, kJ/mol) of benzo(a)pyrene (BaP) formation mechanism starting from benzo(a)anthracene (BaA → BaP) computed at the B3LYP/6-31+G(d,p) [B3LYP] and M06-2X/6-311++G(d,p) [M06-2X] levels of theory. In the latter case, the calculations were performed besides the default integration grid (99 radial shells and 590 angular points per shell) with a finetuned setting as well (99 radial shells and 974 angular points per shell) [M06-2X_gr]. The difference between the activation energies (ΔΔ*G*^‡^*,* kJ/mol) of [M06-2X vs B3LYP] and [M06-2X_gr vs B3LYP] is also tabulated. | | | | | |
| --- | --- | --- | --- | --- | --- |
| **BaA → BaP** | **M06-2X** | **B3LYP** | **M06-2X_gr** | **M06-2X**  vs  **B3LYP** | **M06-2X_gr**  vs  **B3LYP** |
|  | Δ*G*^‡^ | | | ΔΔ*G*^‡^ | |
|  | kJ/mol | | | | |
| a - **TS_ab_** - b | 94.43 | 71.09 | 94.50 | 23.34 | 23.40 |
| c - **TS_cd_** - d | 52.83 | 26.69 | 52.15 | 26.14 | 25.46 |
| d - **Ts_de_** - e | 132.92 | 128.48 | 132.61 | 4.44 | 4.12 |
| e - **Ts_ef_** - f | 114.11 | 116.27 | 114.26 | 2.16 | 2.01 |
| f - **TS_fg_** - g | 51.70 | 27.67 | 51.28 | 24.03 | 23.60 |
| h - **TS_hi_** - i | 72.25 | 48.39 | 72.15 | 23.86 | 23.76 |
| i - **TS_ij1_** - j1 | 120.01 | 104.56 | 121.76 | 15.45 | 17.20 |
| i - **TS_ij2_ -** j2 | 118.63 | 99.64 | 119.71 | 18.99 | 20.07 |
| j1 - **TS_j1k1_ -** k1 | 20.95 | 14.13 | 20.64 | 6.82 | 6.51 |
| j1 - **TS_j1k2_ -** k2 | 27.57 | 21.25 | 27.40 | 6.32 | 6.15 |
| k1 - **TS_k1P_ -** P | 149.04 | 145.39 | 149.83 | 3.65 | 4.43 |
| k2 - **TS_k2P_ -** P | 137.57 | 135.05 | 137.87 | 2.52 | 2.81 |

**Figure S3** and **S4** Transition state structures of benzo(a)pyrene formation starting from chrysene were located at the M06-2X/6-311++G(d,p) level of theory using a finetuned integration grid (99 radial shells and 974 angular points per shell) and depicted along with interatomic distances and bond angle at the reaction site.

| 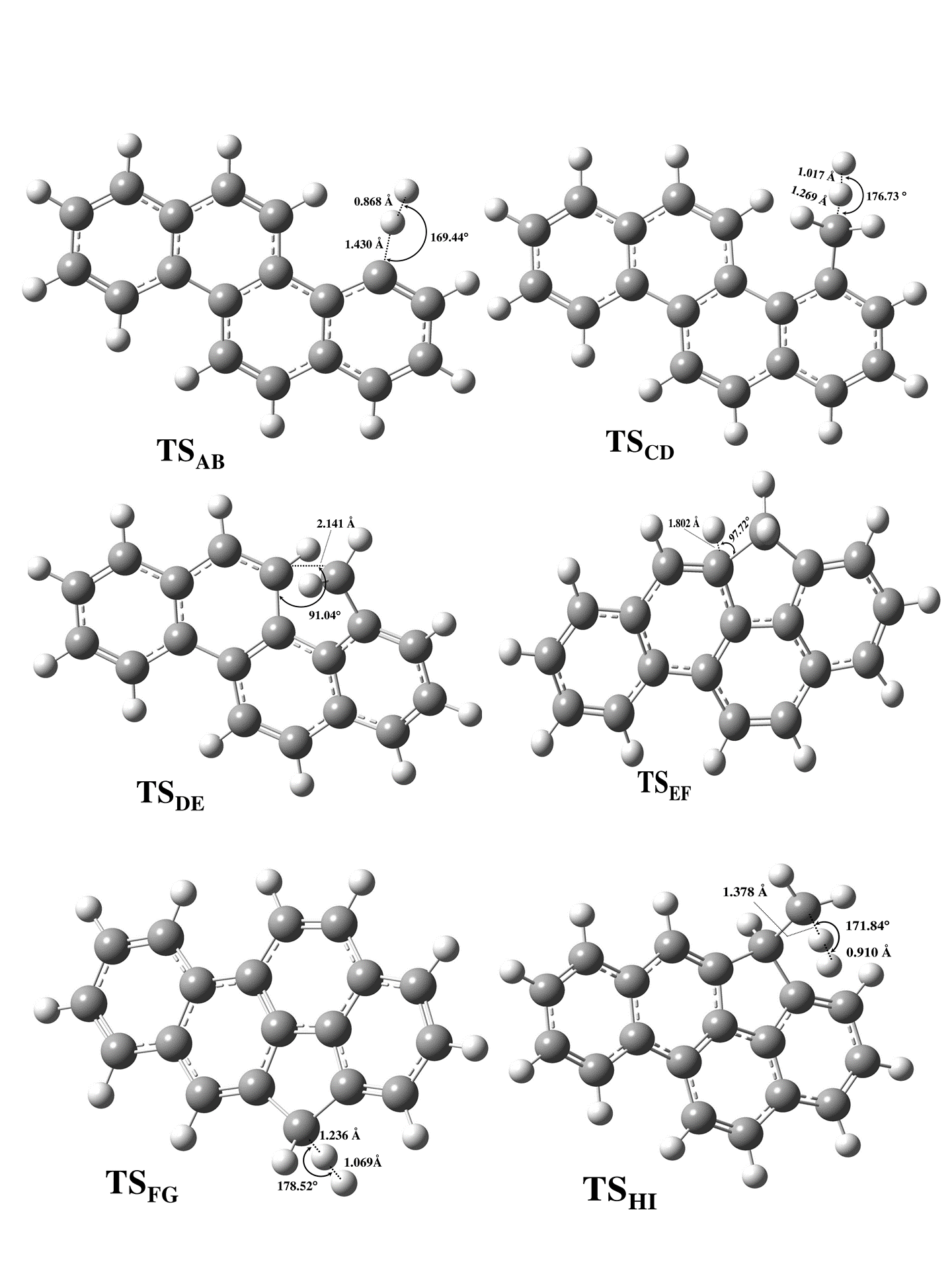 |
| --- |
| **Figure S3** |

| 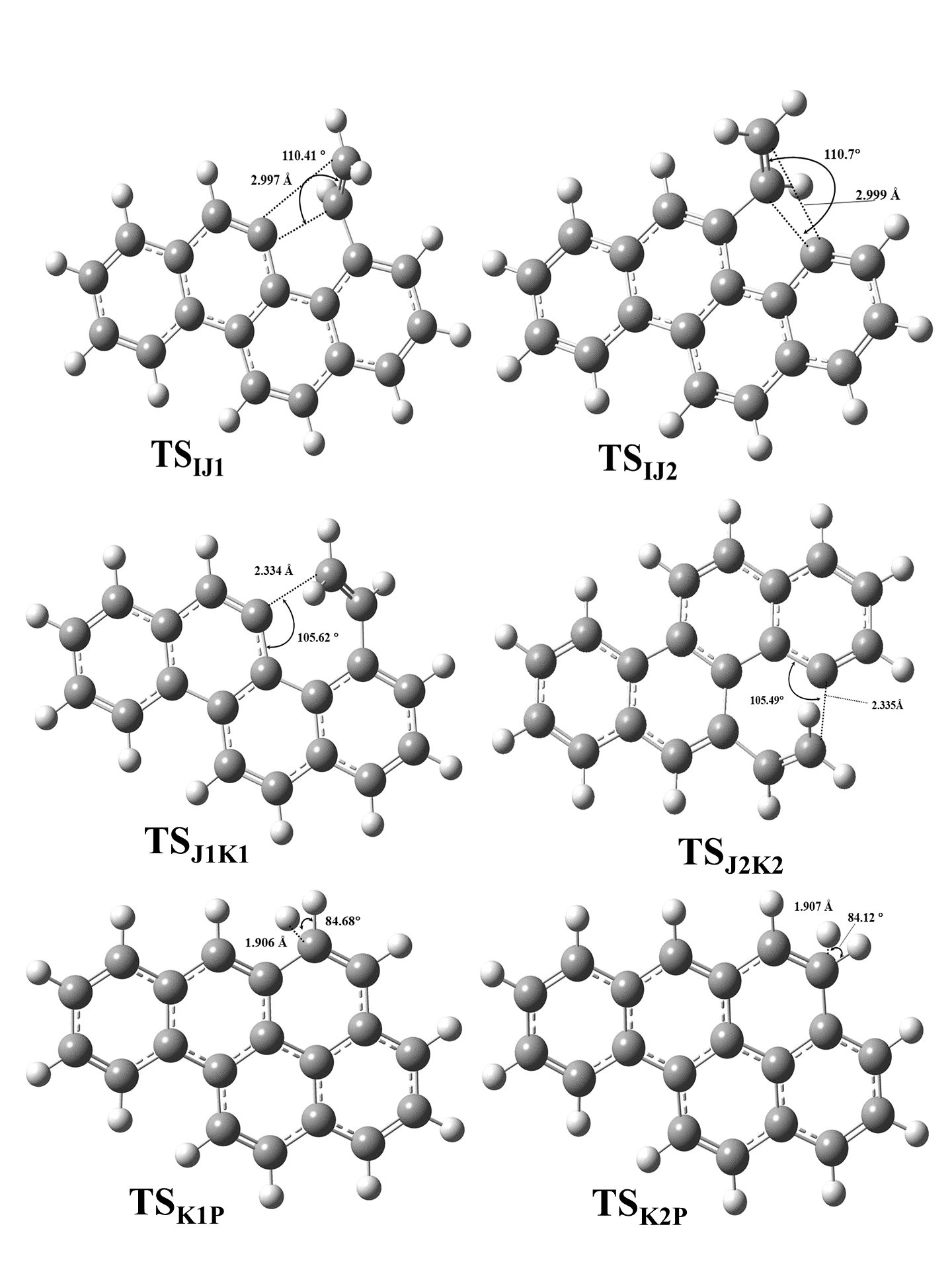 |
| --- |
| **Figure S4** |

**Figure S5** and **S6** Transition state structures of benzo(a)pyrene formation starting from benzo(a)anthracene were located at the M06-2X/6-311++G(d,p) level of theory using a finetuned integration grid (99 radial shells and 974 angular points per shell) and depicted along with interatomic distances and bond angle at the reaction site.

| 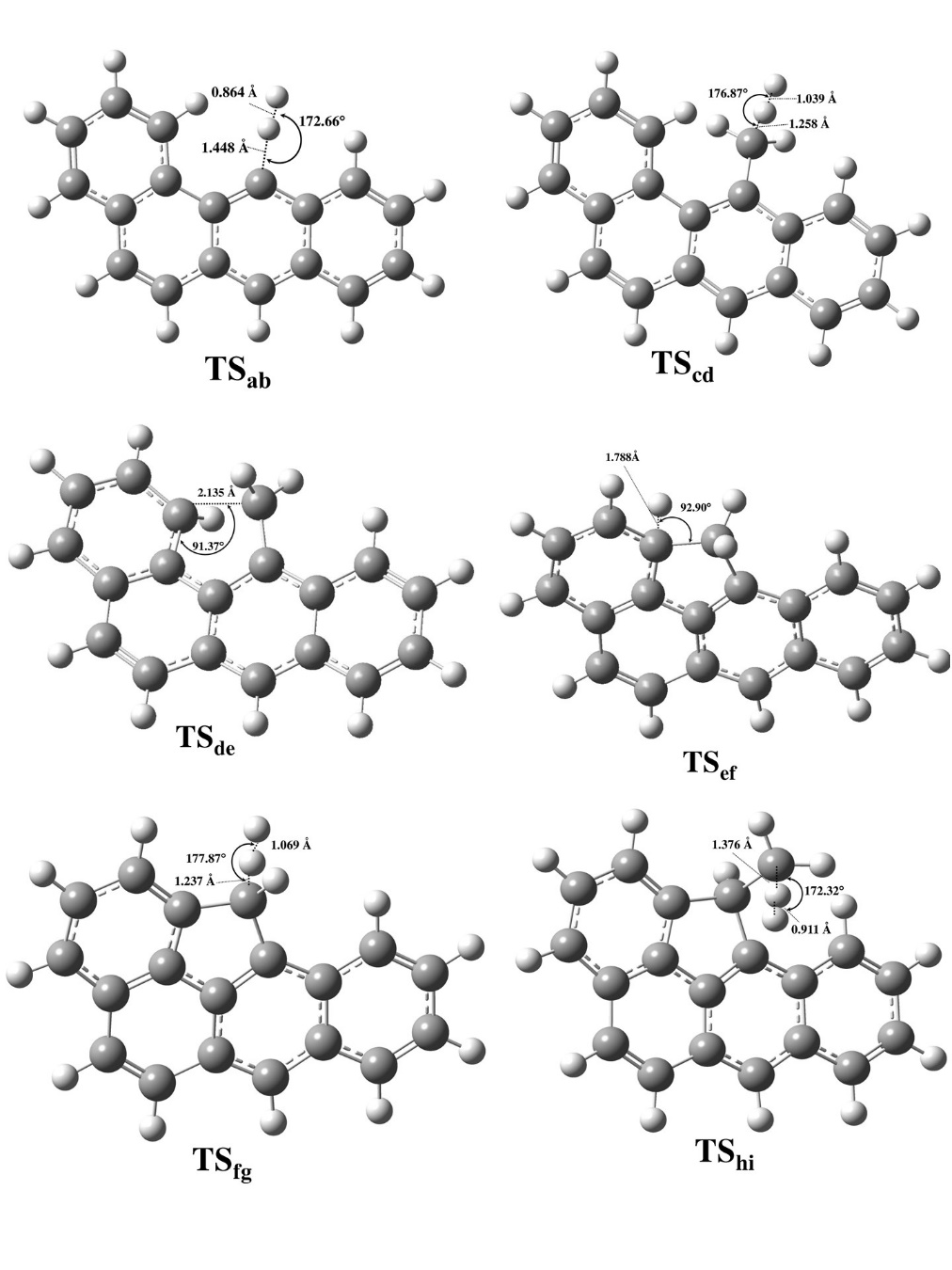 |
| --- |
| **Figure S5** |

| 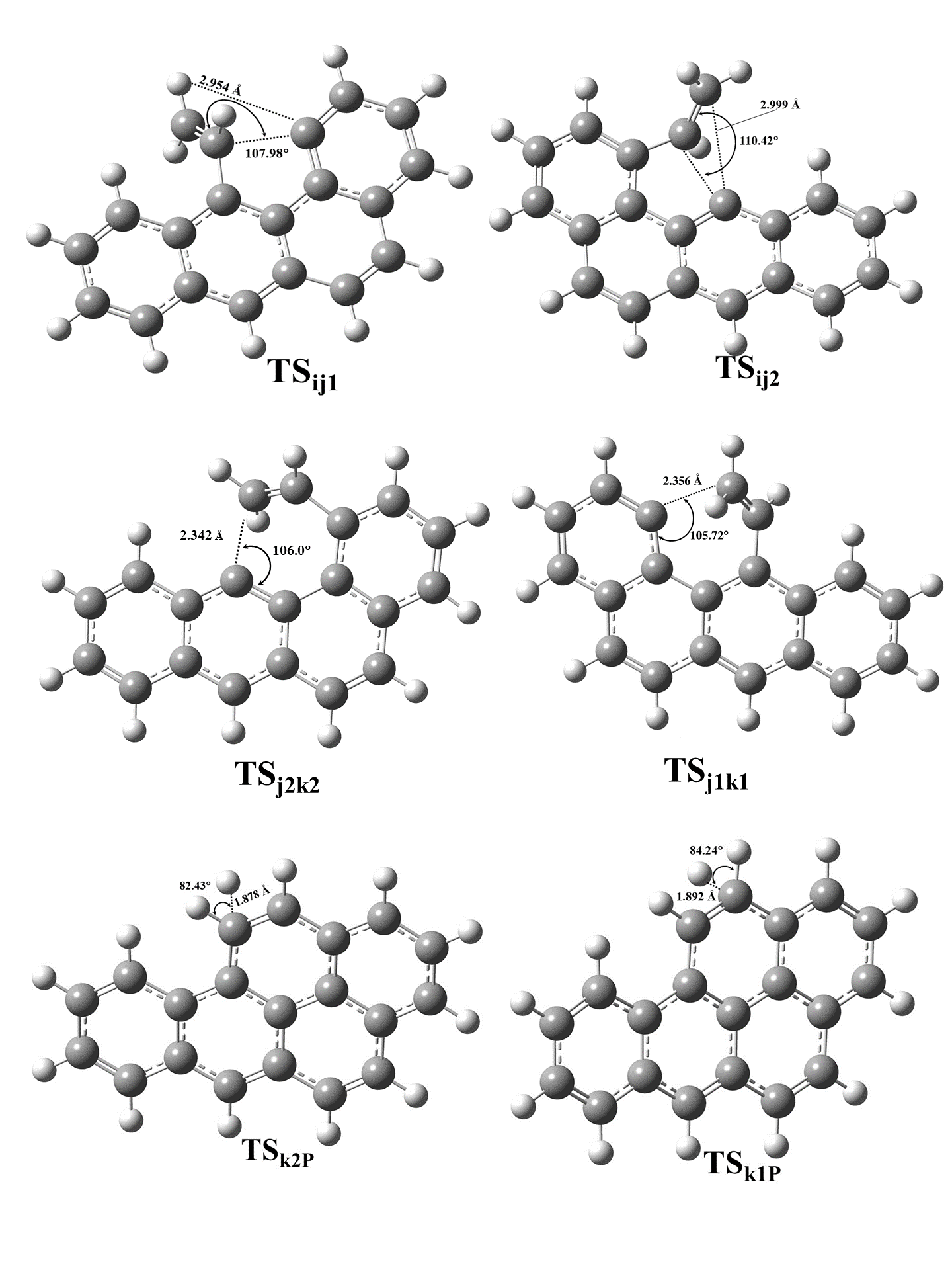 |
| --- |
| **Figure S6** |

**Example input files:**

----------------------------------------------------------------

# M062X/6-311++G(d,p) Int(Grid=99974) Opt Freq

Title

0 2

H 0.00000 0.00000 0.00000

----------------------------------------------------------------

----------------------------------------------------------------

# M062X/6-311++G(d,p) Opt Freq

Title

0 2

H 0.00000 0.00000 0.00000

----------------------------------------------------------------

----------------------------------------------------------------

# B3LYP/6-31+G(d,p) Opt Freq

Title

0 2

H 0.00000 0.00000 0.00000

----------------------------------------------------------------

**References**

1. Burns, D. M., Iball, I. & IUCr. The bond lengths in chrysene. *Acta Crystallogr.* **9**, 314–315 (1956).

2. Gittins, C. M., Rohlfing, E. A. & Rohlfing, C. M. Experimental and theoretical characterization of the S1–S0 transition of benzo[a]pyrene. *J. Chem. Phys.* **105**, 7323 (1998).
